# Neural Reconstruction in Disorders of Consciousness: Prospects and Emerging Challenges

**DOI:** 10.1177/10738584261434939

**Published:** 2026-04-15

**Authors:** Matthew Kolisnyk, Sidath Rankaduwa, Garima Gupta, Karnig Kazazian, Derek B. Debicki, Adrian M. Owen

**Affiliations:** 1 Western Institute of Neuroscience, Western University, London, ON, Canada; 2 Department of Physiology and Pharmacology, Western University, London, ON, Canada; 3 Department of Clinical Neurological Sciences, Western University, London, ON, Canada; 4 Department of Psychology, Western University, London, ON, Canada

**Keywords:** reconstruction, neuroimaging, disorders of consciousness, deep learning, ethics, speech, vision, awareness

## Abstract

Reconstructing mental experience from brain activity is becoming increasingly feasible through advances in neuroimaging and deep learning. Neural signals have been translated into images, text, and speech and have been applied clinically to restore communication and movement in patients with motor paralysis. Extending reconstruction to patients with disorders of consciousness (DoCs) represents the next critical step. DoCs encompass conditions such as the vegetative and minimally conscious states, in which wakefulness is preserved but behavioral signs of awareness are absent, inconsistent, or difficult to interpret. These behavioral signs may not reflect patients’ underlying cognitive capacities, as neuroimaging studies have shown that a subset retains cognitive function. Reconstruction could offer insight into these otherwise inaccessible experiences and potentially restore communication. However, if applied incorrectly, reconstruction risks mischaracterizing a patient’s inner life and compromising their autonomy. To clarify the current landscape, this article reviews the development of reconstruction methods, their emerging clinical applications, and the distinct interpretive challenges associated with applying these approaches to DoCs. It then offers recommendations for evaluating reconstruction results, centered on identifying awareness, validating reconstruction models, and protecting patient autonomy. The aim is to support the responsible advancement of reconstruction and its potential to transform understanding of DoCs.

## Introduction

A fundamental goal of neuroscience is to understand how consciousness emerges from the brain. Researchers have approached this problem by modeling how neural activity maps onto a spectrum of behaviors, actions, and thoughts. As these models increase in complexity, so does the resolution at which the experiences of others can be probed. The process of predicting the current contents of consciousness from neural data is referred to as *reconstruction*. Colloquially described as “mind-reading,” this approach involves building models that translate brain activity into external representations of perception and experience (Kamitani and Tong 2005; Haynes and Rees 2006).

Reconstruction has shown significant clinical promise in restoring functions lost due to brain injury. Some individuals with spinal or brainstem damage, or with amyotrophic lateral sclerosis (ALS), fully retain many cognitive functions (e.g., speech) but lack the motor ability (e.g., speech muscles) to effectively express them. Several so-called neuroprostheses have been developed to address these problems by bypassing motor deficits and decoding intended action directly from brain activity (Silva et al 2024). These devices have enabled patients to translate imagined movement into actual movement, control a computer cursor, and convert inner speech to actual speech (Birbaumer 2006; Hochberg et al 2006; Vansteensel et al 2016; Luo et al 2023; Card et al 2024). Such interventions have profoundly improved patient agency, quality of life, and connectedness with their environment, with several formerly noncommunicative patients reporting directly how these technologies have transformed their lives (Sellers et al 2010; Felgoise et al 2016).

Extending reconstruction to patients with disorders of consciousness (DoCs) presents a far greater challenge. DoCs arise from brain injuries that either abolish consciousness entirely or mask its presence (Jennett and Plum 1972; Owen et al 2006; Giacino et al 2014). Consciousness in this population is generally defined along 2 dimensions, wakefulness (e.g., eye opening, sleep–wake cycling) and awareness (e.g., subjective experience), and is typically evaluated using validated behavioral assessments (Giacino et al 2014; Posner et al 2019). However, neuroimaging studies over the past 2 decades have shown that some patients who appear completely *behaviorally* nonresponsive nonetheless exhibit *neural* evidence suggestive of a rich inner experience (Owen et al 2006; Coleman et al 2007; Pan et al 2020; Norton et al 2023; Bodien et al 2024). Because reconstruction provides an unprecedented means of decoding perceptual and cognitive content directly from brain activity, as well as the potential to restore speech, there is growing interest in applying it to patients with DoCs (Fischer et al 2025).

Despite this growing interest, the misapplication of reconstruction in DoCs carries substantial risks of misuse, misinterpretation, and misappropriation. Currently, there is no roadmap for interpreting what a reconstructed output does or does not reveal about a patient’s subjective experience. For example, if a model consistently reconstructs faces from a patient’s brain activity, does this indicate conscious perception or simply residual visual encoding? By analogy, it has been shown that speech generates a pattern of brain activity in deeply anaesthetized controls that is highly similar to that seen in healthy, awake individuals, yet their subjective experience of “hearing” or “comprehending” is undoubtedly quite different (Davis et al 2007; Milinkovic et al 2025). This problem is amplified by how reconstruction models are designed. These models constrain outputs to conform to the statistical regularities of their training data or to typical patterns of healthy cognition, which likely differ from the patient’s own perception. Speech reconstruction models, for example, enforce the grammatical rules of language, despite there being no guarantee that this reflects the patient’s true inner speech (e.g., if the patient is aphasic). Ultimately, the danger is that reconstructed outputs may bear little or no relation to the patient’s actual experience, if any exists at all, risking the misattribution of awareness and misrepresentation of a patient’s inner life.

To better understand these issues, this article first examines the foundations and evolution of reconstruction, tracing its development from early decoding studies to recent advances in speech and vision models. The current clinical applications of reconstruction are considered next, particularly in the context of restoring communication in motor-impaired patients. Then the article turns to the philosophical, methodological, and interpretive challenges that complicate the application of reconstruction in DoCs. Finally, the article concludes by outlining principles and practical considerations intended to guide the development and interpretation of reconstruction models, so that future applications reflect patients’ experiences with fairness, accuracy, and care.

## What Is Reconstruction?

**Figure 1.**
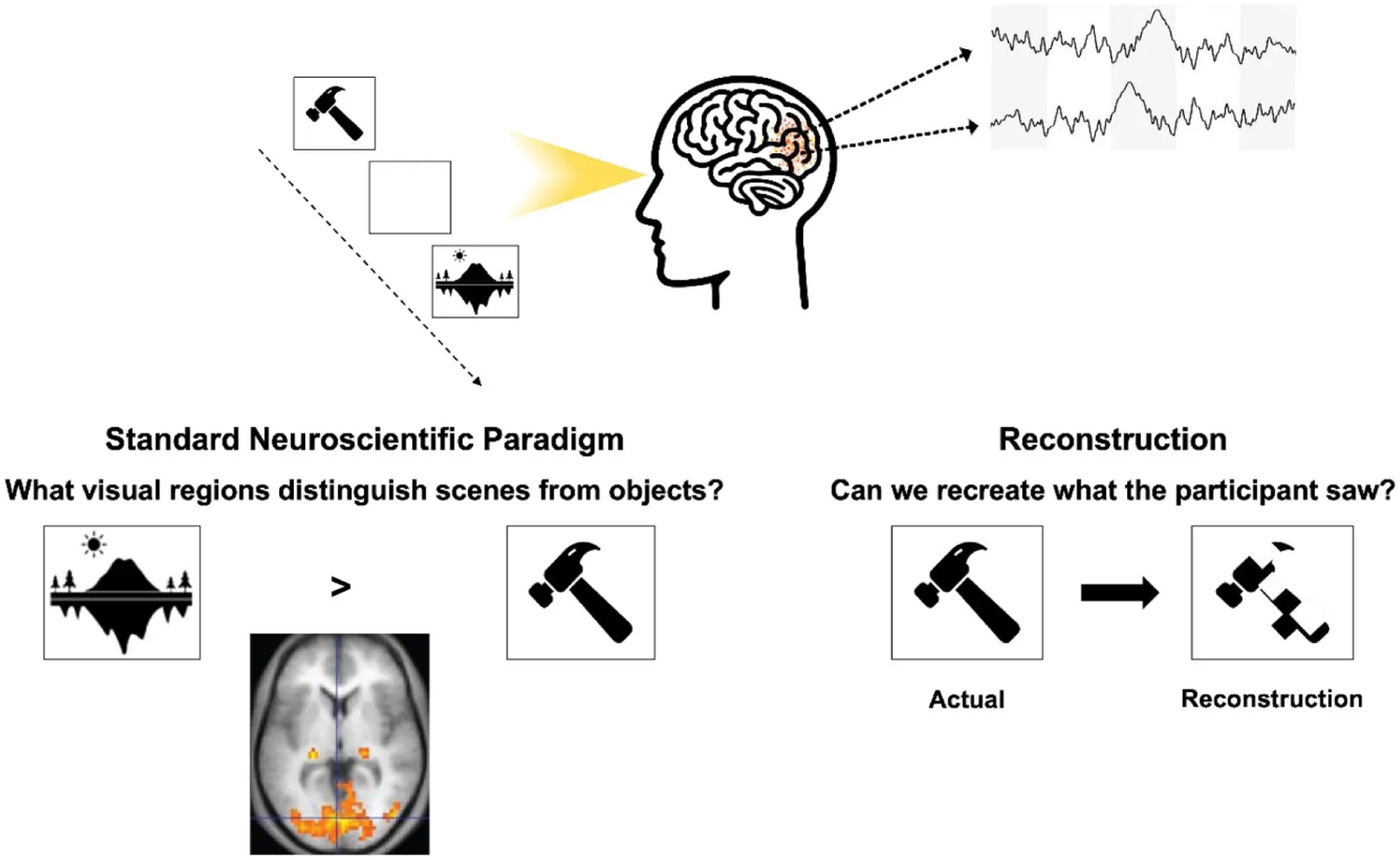
A schematic illustrating key differences in the approach used by standard experimental neuroscience paradigms and reconstruction-based approaches. In a typical visual neuroscience study, participants view objects or scenes while researchers identify the brain regions that respond to those stimuli. In contrast, reconstruction aims to re-create the specific object or face being viewed directly from measured brain activity. One implementation, for example, reduces each image to a 20-pixel × 20-pixel grid and trains a model to predict the RGB value of each pixel based on neural signals recorded during viewing.

Reconstruction differs from standard experimental approaches in neuroscience, which typically aim to identify brain regions activated by specific stimuli or experimental manipulations. Instead, reconstruction inverts this relationship: it builds models that convert neural activity into predictions about the stimulus or mental content that produced it (see Figure 1 for an example from visual neuroscience). This approach has a long history within the field of decoding (Haynes and Rees 2006; Kriegeskorte and Douglas 2019) and classification (Haxby et al 2001) but often has focused on binary or category-level output prediction. Advances in deep learning have enabled richer mappings between neural activity and stimulus features, allowing reconstruction of perceptual content at a level of detail previously unattainable. The ultimate goal of reconstruction is not merely to classify activity or retrieve familiar examples but to approximate the participant’s experience using information measured directly from the brain.

### Tracing Improvement in Reconstruction Capability

The origins of reconstruction began with efforts to map raw brain signals to simple, often binary, representations of movement or cognition. Early electroencephalography (EEG) systems translated scalp-recorded electrical activity into basic commands, enabling letter selection for communication (Birbaumer et al 1999), control of a cursor in 8 directions (Wolpaw et al 2004), and coarse movements of prosthetic arms (Wessberg et al 2000; Taylor et al 2002; Hochberg et al 2006). With the widespread adoption of functional magnetic resonance imaging (fMRI), which tracks slow changes in blood oxygenation, researchers extended these methods to include visual tasks, enabling them to differentiate between a limited set of objects, orientations, rotations, and colors (Haxby et al 2001; Kamitani and Tong 2005).

While these early systems demonstrated feasibility, they faced major constraints in both speed and resolution, limiting their clinical utility. For example, the Birbaumer et al (1999) study required extensive training: 2 patients with ALS learned to generate a positive electrical inflection in response to prompts. Each inflection, lasting 4 to 6 s, progressively split the alphabet in half until a single character remained. One participant required 16 h to compose a message (roughly 2 characters per minute). Similarly, Wolpaw et al (2004) showed that cursor movements could be reliably predicted, but control was limited to just 8 possible locations. In the domain of fMRI, early studies were restricted to distinguishing between 6 simple dot patterns or 4 highly distinct object categories (Haxby et al 2001; Thirion et al 2006). While groundbreaking for their time, they fell short of early “mind-reading” claims, which felt more speculative than achievable (Naselaris et al 2011).

**Figure 2.**
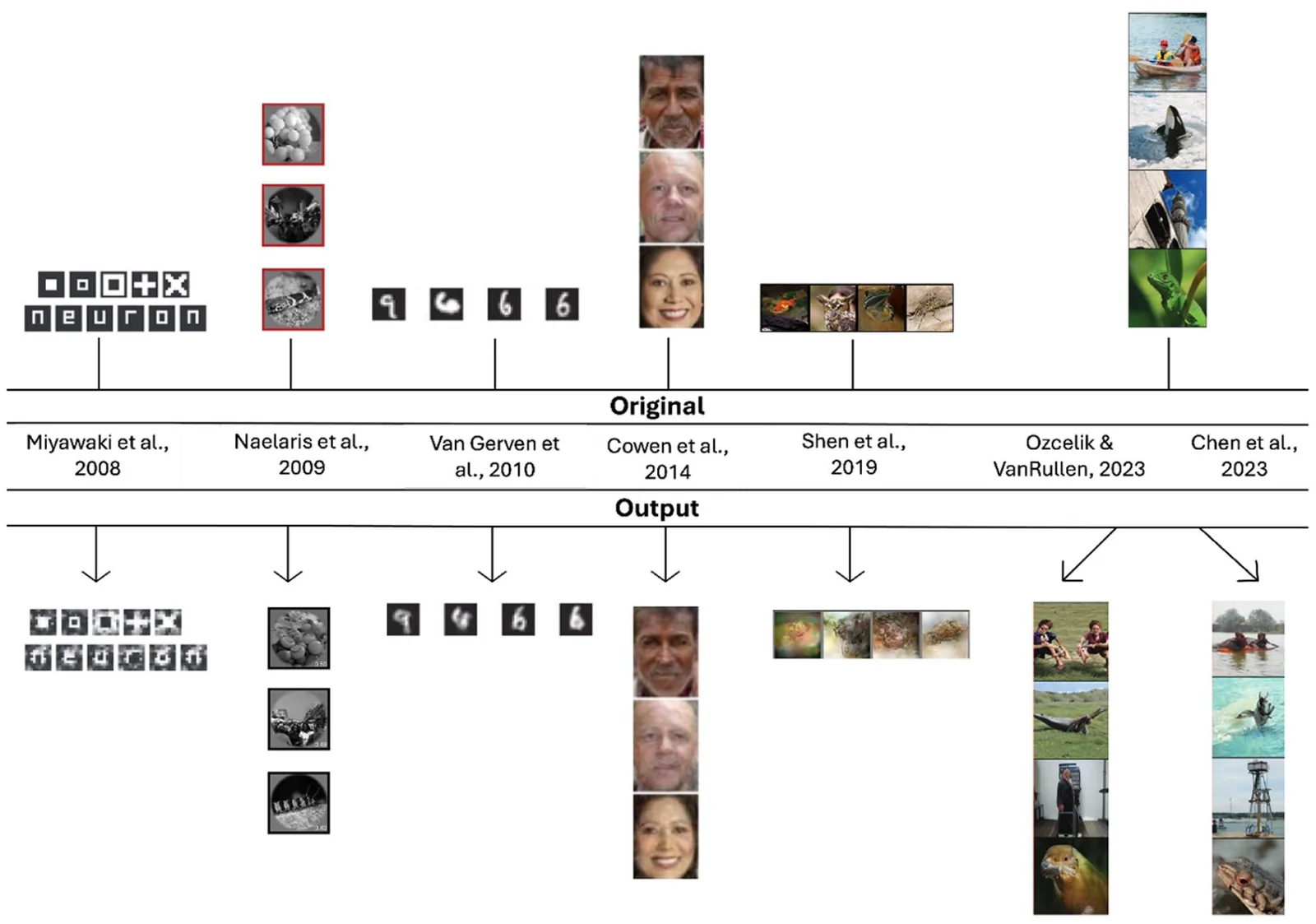
Development of visual reconstruction over time. The top row (“Original”) shows the stimuli presented to participants, and the bottom row (“Output”) shows the images generated from neural activity using the respective methods. Early studies (e.g., Naselaris et al 2009; Van Gerven et al 2010) relied on classification or selecting the closest match from a predefined set of images. Subsequent approaches introduced deep learning–focused approaches (e.g., Shen et al 2019; Chen et al 2023; Ozcelik and VanRullen 2023), enabling more detailed and flexible outputs. Chen et al (2023) and Ozcelik and VanRullen (2023) used the same stimulus set. Elements adapted from previously published work (see figure for citations), with permission where required.

In recent years, the fidelity of reconstructed outputs has improved to the point that the concept of “mind*-*reading” is harder to dismiss (see Figure 2 for examples of advances in visual reconstruction). These gains have been driven by several factors, including enhanced recording devices, improvements in neural signal processing, and a better understanding of how specific brain regions map onto cognitive functions. However, the most transformative changes have come from advances in deep learning.

**Figure 3.**
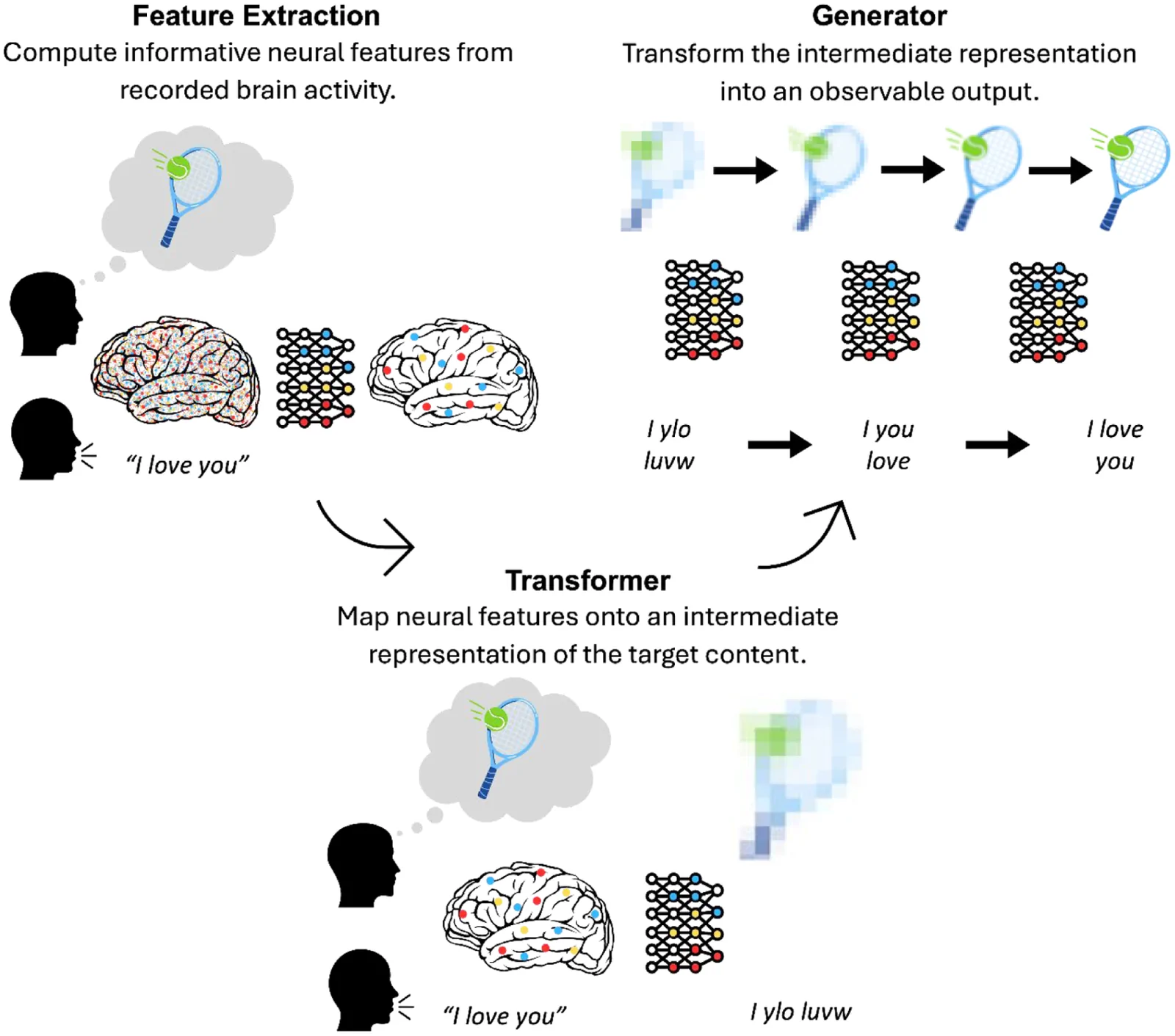
Simplified schematic of the multistep reconstruction process. First, neural signals are preprocessed and transformed into informative neural features. Next, a decoding model (the transformer) maps these features onto an intermediate representation of the reconstructed content, serving as a bridge between brain activity and output. Finally, a generative model (the generator) transforms this intermediate representation into an observable image or sentence.

Reconstruction methods have been developed across many sensory and motor domains, including vision, touch, audition, and movement, but advances have been most pronounced in vision and speech (for detailed reviews, see Silva et al 2024; Kamitani et al 2025). Broadly, these state-of-the-art reconstruction methods (e.g., Shen et al 2019; Moses et al 2021; Chen et al 2023; Ozcelik and VanRullen 2023; Card et al 2024) have relied on multistep deep learning pipelines that transform brain activity into increasingly structured representations (see Figure 3).

First, raw brain signals are preprocessed and restricted to regions of interest. From these signals, neural features are extracted to minimize redundancy and capture informative aspects of brain activity. These features may reflect temporal dynamics (e.g., spike timing or oscillatory structure), spatial activation patterns (e.g., multivoxel responses), or network-level properties (e.g., connectivity). The choice of features, anatomical location, and temporal scale determines what information is preserved and therefore constrains what can ultimately be reconstructed.

Next, a decoding model, the transformer, maps these neural features onto an intermediate representation of the reconstructed content. This representation serves as a bridge between neural activity and the final output. In speech reconstruction, neural activity may be translated into linguistic units such as phonemes or characters (Moses et al 2021; Willett et al 2021; Card et al 2024). In early visual reconstruction approaches, neural features were mapped onto spatial filters or image bases (Miyawaki et al 2008; Cowen et al 2014). Contemporary methods increasingly map neural activity into the internal feature spaces of large-scale deep learning models (Shen et al 2019; Chen et al 2023; Ozcelik and VanRullen 2023). These models, trained on millions of examples and parameterized by millions of weights (Deng et al 2009; Krizhevsky et al 2012; Simonyan and Zisserman 2015), represent stimuli as patterns of activity across high-dimensional feature spaces, often referred to as latent spaces. These intermediate representations encode information spanning low-level perceptual attributes through object- and category-level structure. Although developed within computer vision and natural language processing, early architectures were loosely inspired by hierarchical principles observed in the brain (LeCun et al 1989, 1998), and subsequent analyses have demonstrated partial correspondence between these hierarchical features and neural representations (Güçlü and Gerven 2014; Zeiler and Fergus 2014; Horikawa and Kamitani 2017). Mapping neural features onto these intermediate representations therefore provides a high-dimensional, structured description of stimulus or mental content that enables reconstruction.

**Figure 4.**
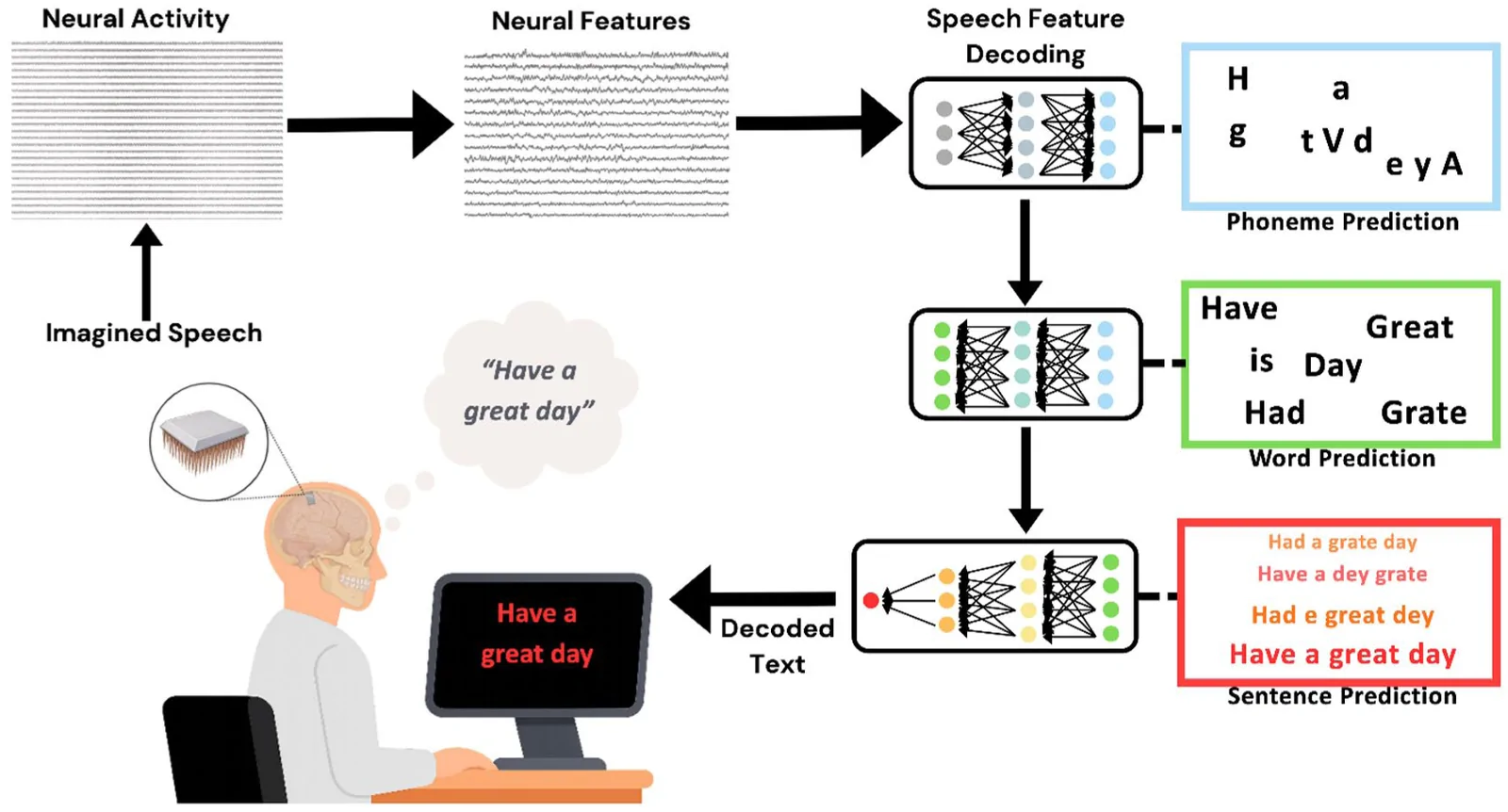
Traditional speech reconstruction pipeline. First neural activity is recorded, typically directly using electrode arrays placed on motor speech areas. The neural activity is condensed or converted into meaningful features and associated with lower-level aspects of speech, such as phonemes. The reconstruction model then takes the phoneme predictions through word- and sentence-level predictions before the final output is presented on a screen.

Once this intermediate representation has been estimated, a generative model, the generator, transforms the latent description into an observable output. In speech reconstruction, hierarchical language models trained on large text databases convert predicted linguistic features into coherent words and sentences. This can be thought of as occurring in a stepwise fashion: transforming phonemes into characters, characters into words, and words into sentences according to learned statistical regularities in natural language (Moses et al 2021; Willett et al 2021; Card et al 2024; see Figure 4). In visual reconstruction, state-of-the-art generative architectures such as generative adversarial networks (GANs) or diffusion models synthesize images consistent with the predicted latent features (Shen et al 2019; Chen et al 2023; Ozcelik and VanRullen 2023). GAN-based approaches generate images through competition with a discriminator network (Goodfellow et al 2014), whereas diffusion models iteratively transform noise into structured images conditioned on the intermediate representation (Dhariwal and Nichol 2021). This stage optimizes and realizes the intermediate representation into a genuine reconstructed output.

Modern reconstruction thus involves translating neural features into an intermediate representation and rendering that representation into an observable image or sentence. While not the focus of this overview, contemporary standards further require careful dataset selection, rigorous evaluation procedures, and the ability to generalize to previously unseen stimuli (e.g., zero-shot prediction) to prevent the possibility of spurious reconstruction (Kamitani et al 2025; Shirakawa et al 2025). The implications of these considerations are especially important in DoCs and are discussed in later sections.

## Clinical Applications of Reconstruction

These recent successes have enabled clinical applications of multistage reconstruction models. They currently span diverse domains, from restoring movement and sensation to enabling communication through text, speech, or facial expression (Ajiboye et al 2017; Friedenberg et al 2017; Cajigas et al 2021; Singer-Clark et al 2025). Among these developments, advances in speech reconstruction have been particularly transformative, given the central role of speech in personal identity, agency, and meaningful connection with others. Because of its potential relevance to future applications in DoCs, speech reconstruction will serve as the focus of the next section.

### Reconstructing Language in Clinical Populations

Several early studies sought to develop brain-based methods for faster and more accurate communication. One solution was to reconstruct imagined written letters directly from neural activity. For example, Willett et al (2021) asked a quadriplegic patient to imagine writing individual characters from sentences while 2 surgically implanted microelectrode arrays recorded electrical activity from the “hand knob” region of the precentral gyrus. Neural data were collected during imagined writing of over 30,000 characters. A recurrent neural network, a deep learning model designed to capture temporal dependencies in sequential data, was trained to map electrical activity onto individual characters. During final testing, typing speeds reached 90 characters per minute with a mean character error rate of 5.4%. When a secondary language model was incorporated to capture the statistical structure of human language, the error rate decreased further to 0.89%.

Around the same time, Moses et al (2021) introduced a neuroprosthesis for speech in a patient with anarthria and spastic quadriparesis following a brainstem stroke. High-density microelectrode arrays were implanted over the speech sensorimotor cortex, and attempted speech was decoded from the resulting neural activity. A recurrent neural network was trained on sentences drawn from a fixed 50-word vocabulary and tested as the patient attempted to speak new sentences displayed on a screen. The neural-only model yielded a word error rate of 60.5%, whereas integration with a language model reduced this to 25.6% and enabled unprompted communication at 15.2 words per minute. Although more error-prone than the method used by Willett et al (2021), this approach allowed attempted speech at the word level rather than imagined character-based writing.

Word-level reconstruction based on attempted speech was intuitive and efficient, yet restricting the patient to a 50-word vocabulary limited the range of thoughts that could be expressed. Moreover, the requirement of attempted speech excluded individuals with paralysis of the muscles required for articulation. To address these limitations, Metzger et al (2022) decoded individual “code words” from silent spelling attempts in a patient with severe limb and vocal tract paralysis. These code words (e.g., “alpha”) corresponded to individual letters (e.g., “a”) that were combined to form sentences in real time. As in earlier studies, neural signals were recorded from speech and adjacent sensorimotor cortices. The predicted characters were processed through 2 sequential language models, one that constructed words from letters and another that generated plausible sentences. Although slower and less accurate than previous systems, this approach supported a significantly larger vocabulary of 1152 words, achieving a median character error rate of 6.13% at 29.4 characters per minute.

The principal motivation behind speech neuroprostheses is to restore communication, yet several recent studies have focused instead on reconstructing other essential components of speech, such as tone and expressive control. For instance, Angrick et al (2024) decoded 6 phrases and synthesized them in the patient’s preinjury voice. Similarly, Metzger et al (2023) reconstructed speech from decoded text and used a facial avatar selected by the patient to animate speech with realistic facial movements. Both studies demonstrated the growing use of reconstruction to create more human-like, personalized interactions.

These studies culminated in recent work by Card et al (2024), who developed a fast and accurate neuroprosthesis capable of rendering speech in the patient’s preinjury voice. The study involved a 45-year-old individual with severe dysarthria and quadriparesis due to ALS. A key innovation in this model was its focus on reconstructing phonemes, which were then passed to secondary language models to construct coherent words and then sentences. On the first day of use, the neuroprosthesis required only 30 min of calibration data to achieve 99.6% accuracy with a constrained 50-word vocabulary. Following additional training on a 125,000-word vocabulary, accuracy reached 90.2%. Continued optimization enabled real-time text output and conversational speech at an average rate of 32 words per minute, which the system vocalized in the participant’s pre-ALS voice.

Within the span of 5 years, advances in neurotechnology, deep learning, and language modelling have enabled the conversion of neural speech signals into audible speech sufficiently natural to support everyday communication. Their success raises a pressing question: how should reconstruction proceed when residual speech, or awareness itself, may not be present?

## Residual Functioning in Patients with Disorders of Consciousness

DoCs encompass a spectrum of neurological impairments that follow severe brain injury and disrupt the mechanisms underlying wakefulness and awareness (Owen et al 2006; Giacino et al 2014). The degree to which these dimensions are preserved varies across diagnostic categories. In coma, both wakefulness and awareness are absent. In the unresponsive wakefulness syndrome (also known as the vegetative state), wakefulness is preserved, but awareness is lacking. By contrast, patients in a minimally conscious state show intermittent yet reproducible signs of awareness.

Behavioral assessment remains the gold standard for diagnosing DoCs (Giacino et al 2014). However, because of factors such as fluctuating arousal, spontaneous or inconsistent behavior, and sensory or motor impairments, behavioral diagnosis is unreliable and may fail to capture the full extent of preserved cognitive function (Schnakers et al 2009). As a result, functional neuroimaging methods such as fMRI and EEG have increasingly been used to measure brain function directly.

In a landmark fMRI study, Owen et al (2006) demonstrated willful, instruction-dependent neural responses in a behaviorally unresponsive patient previously diagnosed as being in a vegetative state. When instructed to imagine playing tennis, the patient activated supplementary motor areas, whereas when the patient was instructed to imagine walking through their home, parahippocampal and posterior parietal regions were engaged. Neither response was present at rest. This dissociation between behavioral presentation and neural evidence indicates that some patients retain complex cognitive function despite the absence of behavioral responsiveness. Adaptations of this paradigm later enabled binary communication through motor imagery to signal “yes” and spatial imagery to signal “no,” allowing patients to answer simple questions (Monti et al 2010). These findings provide compelling evidence that awareness may persist undetected in some patients and that a wider range of cognitive function may be preserved than previously believed. Recent estimates suggest that approximately 15% to 25% of behaviorally unresponsive patients demonstrate evidence of preserved awareness (Kondziella et al 2016; Bodien et al 2024).

Still, the form that awareness takes in these individuals, and DoCs at large, remains unclear. Moreover, patients who fail to follow commands may not necessarily lack conscious experience, as deficits in language comprehension, working memory, or executive function can produce false-negative results. At the same time, “islands” of preserved cognitive function may persist amid severe global impairment (Bayne et al 2020). Over the past 2 decades, subsets of patients with DoCs have shown evidence of intact auditory, visual, or somatosensory processing (Coleman et al 2007; Monti et al 2010; Naci et al 2014; Calabrò et al 2021; Norton et al 2023), and some exhibit higher-order functions such as selective attention (Pan et al 2020) or narrative processing during naturalistic movie viewing (Naci et al 2014; Laforge et al 2020).

Nevertheless, these demonstrations still provide only a limited glimpse into patients’ subjective experiences. Reconstruction offers a promising means of probing subjective states across the diagnostic range of patients with DoCs with far greater granularity and scale than before. However, reconstruction research must also navigate critical ethical and clinical considerations to safeguard patient welfare and manage caregiver expectations (Peterson et al 2021; Young et al 2024). The following section addresses the philosophical and methodological challenges that must be considered before reconstruction can be responsibly applied in this patient group.

## Considering Reconstruction Results in Patients with Disorders of Consciousness

### Hypothetical Case

To illustrate the challenges of interpreting reconstruction outputs, consider a hypothetical patient with DoC, SK (Box 1).

**Box 1.** Visual Reconstruction in a Patient with Disorder of Consciousness—Hypothetical Case.

*SK is a 38-year-old man who sustained a traumatic brain injury in a motor vehicle collision 15 years ago. When admitted to intensive care, he was behaviorally unresponsive, and his prognosis for recovery was considered poor by the health care team based on clinical factors. His family agreed to continue supportive management and not to withdraw life-sustaining measures. Over several weeks, SK gradually regained the ability to open his eyes and withdraw his hand in response to painful stimuli, but he remained unable to follow behavioral commands (e.g., hand squeezing, object grasping, sustained eye fixation, or communication). Because he demonstrated wakefulness (eyes open between periods of sleep) without clear signs of awareness, he was diagnosed with unresponsive wakefulness syndrome.* *After transfer to a long-term supportive care facility, SK never developed consistent clinical evidence of awareness. However, his family occasionally reported behaviors they interpreted as signs of responsiveness, such as prolonged eye contact, occasional squeezing of their hands on command, or making brief vocalizations when spoken to. When they learned that some patients with DoCs exhibit covert awareness detectable only through neuroimaging, they contacted a specialized research center to explore additional testing.* *Given SK’s eye-opening combined with his lack of reliable behavioral responses, a decision was made to look for any signs of retained visual experience using state-of-the-art reconstruction algorithms. The goal was to determine whether images reconstructed from SK’s brain activity resembled the visual stimuli presented to him. While in an fMRI scanner, SK viewed a range of stimuli, including faces, houses, tools, and emotionally salient images within these categories (e.g., familiar versus unfamiliar faces).* *The research team employed a multistage deep learning model consistent with prior literature. First, they extracted relevant fMRI features from canonical visual processing regions. These features were then passed to a transformer model to generate an intermediate visual representation, which was refined by a generator model to create a realistic output. The final reconstruction consisted of a 50 × 50 matrix of RGB values, enabling visualization of the predicted image. Remarkably, several reconstructions closely resembled the original stimuli.* *These results had a profound emotional impact on SK’s family, especially when they saw reconstructed images of their own faces. To them, this confirmed their long-held belief that SK remained aware and that the reconstructions provided a direct window into his subjective experience.* *However, the clinicians who received these reconstructed outputs remained uncertain about whether they indicate that SK was having a conscious experience or, rather, that his brain was processing these objects automatically, in the absence of awareness. Moreover, the research team was uncertain whether the reconstructed outputs directly reflected SK’s inner experience or were confabulated by the generator model.*

Although this example focuses on visual reconstruction, similar interpretive issues arise across modalities. Broadly, these concerns fall into 2 categories: 1) the difficulty of inferring conscious experience from neural activity and 2) the tendency to overlook constraints imposed by reconstruction models.

### Encoding ≠ Awareness

The central question is whether SK’s reconstructed outputs reflect conscious perception or merely the residual neural infrastructure necessary to encode facial stimuli. Studies of unconscious face processing show that briefly presented or masked faces reliably activate visual cortical regions, including the fusiform cortex, even when participants are unaware of the stimuli (Axelrod et al 2015). A recent meta-analysis found that both conscious and unconscious face processing engage early visual areas, whereas only conscious perception consistently recruits higher-order regions such as the inferior frontal gyrus, intraparietal sulcus, and anterior cingulate cortex (MacLean et al 2023). Importantly, visual reconstruction algorithms can generate high-fidelity outputs using signals from early visual regions alone, with minimal contribution from higher-order areas (Cowen et al 2014; Figure 2). Finally, in the first case of its kind, face-specific activity in the fusiform gyrus was observed in a patient diagnosed as vegetative, yet later reports (when the patient recovered) suggest that this was not accompanied by any conscious experience of seeing faces (Menon et al 1998). Thus, the presence of an accurate reconstruction demonstrates encoding but does not establish conscious perception. In SK’s case, it remains plausible that a reconstructed face could still arise solely from unconscious processing.

### Linking Empirical Findings to Theories of Consciousness

This problem is compounded by longstanding theoretical debates about the neural basis of consciousness. According to some theories, SK’s visual activity likely reflects only unconscious encoding, whereas others suggest it could indicate genuine perception. The global neuronal workspace theory (Baars 2005; Dehaene and Changeux 2011; Mashour et al 2020) proposes that sensory representations become conscious only when they are globally broadcast to frontoparietal systems, making activity confined to early visual cortex insufficient evidence of awareness. In contrast, recurrent processing theory (Lamme 2020) and integrated information theory (Tononi 2004) suggest that recurrent or sustained activity within posterior regions may be sufficient to generate conscious experience (Boly et al 2017). On this basis, attributing conscious awareness to reconstructed outputs lacks a clear theoretical foundation.

### The Philosophical Challenge

Even if empirical research were to identify reliable neural correlates of conscious perception, a deeper problem is explaining why any neural process is accompanied by subjective experience at all. This is the essence of the hard problem of consciousness (Chalmers 1995). The so-called easy problem involves identifying the neural mechanisms that underlie behavior and perception, including mapping patterns of brain activity to specific sensory inputs or reconstructed stimuli. These questions are considered “easy” because they concern functional relationships that can be observed and measured. The hard problem, however, asks why any pattern of neural activity gives rise to qualitative, subjective experience. A reconstruction model may accurately predict a stimulus from brain activity, yet this does not demonstrate that the patient is consciously experiencing it. Reconstruction therefore does not bridge the explanatory gap between neural activity and conscious perception (Levine 1983).

### Technical Concerns

Beyond neuroscientific and philosophical concerns, recent improvements in reconstruction performance may reflect factors other than the brain signals themselves. Gains in accuracy often depend less on the quality of the underlying neural data or the mapping between that activity and the intermediate representation, and more on how models transform these representations into convincing naturalistic outputs. For example, visual reconstruction improved substantially when brain-based predictions were refined using stimulus-specific semantic and higher-order visual features (Naselaris et al 2009) and improved further with the introduction of generative models that transform intermediate representations into naturalistic images (Chen et al 2023; Ozcelik and VanRullen 2023). Similar concerns arise in speech neuroprostheses, where language models convert noisy neural predictions into plausible words and coherent sentences (Silva et al 2024). In many cases, language modeling has transformed systems with limited clinical utility (e.g., 1 character per minute; Chaudhary et al 2022) into functionally usable communication devices (e.g., 32 words per minute; Card et al 2024). However, recent critiques suggest that apparent improvements in reconstructed outputs may, in some cases, reflect a combination of classification and model-driven hallucination rather than true generalization beyond the training data (Shirakawa et al 2025). As a result, increasingly realistic outputs do not necessarily indicate more informative neural decoding but may instead reflect the growing influence of model-imposed structure.

These challenges are not confined to the final generator stage. Even at the transformer step, models may generate outputs that appear valid but are based on computations fundamentally different from those used by the brain. Deep neural networks are universal function approximators, meaning they can map inputs to outputs using any mathematical transformation that minimizes error (Kriegeskorte 2015). This flexibility is acceptable for engineering applications, where only the quality of the final output matters. In neuroscience and clinical care, however, a reconstruction may appear convincing even if it was generated through a function no brain would use. Without careful validation, such outputs can easily be mistaken for evidence of conscious experience.

These considerations matter greatly for patients with DoCs. In this context, the objective is not merely to generate plausible outputs but to represent a patient’s inner experience faithfully. While generator models may appropriately refine intermediate representations when cognitive functions are known to be preserved, the same assumption is far riskier when awareness may be minimal or absent. Because many reconstruction systems are trained to reproduce the statistical structure of natural images or language corpora, a compelling output may reflect internal model regularities, or model hallucination, rather than brain-derived information (Shirakawa et al 2025). If models primarily impose structure on outputs, they risk misrepresenting the character of a patient’s experience. Technologies intended to give patients a voice may instead substitute one shaped by model-based assumptions. Moreover, current systems cannot reconstruct the absence of experience, which in some cases may be the most accurate reflection of a patient’s state. Responsible application in DoCs therefore requires methods that approximate intrinsic neural computation and incorporate explicit safeguards against spurious reconstruction, so that a patient’s authentic experience is preserved.

## Interpreting and Clarifying Reconstruction Outputs

**Figure 5.**
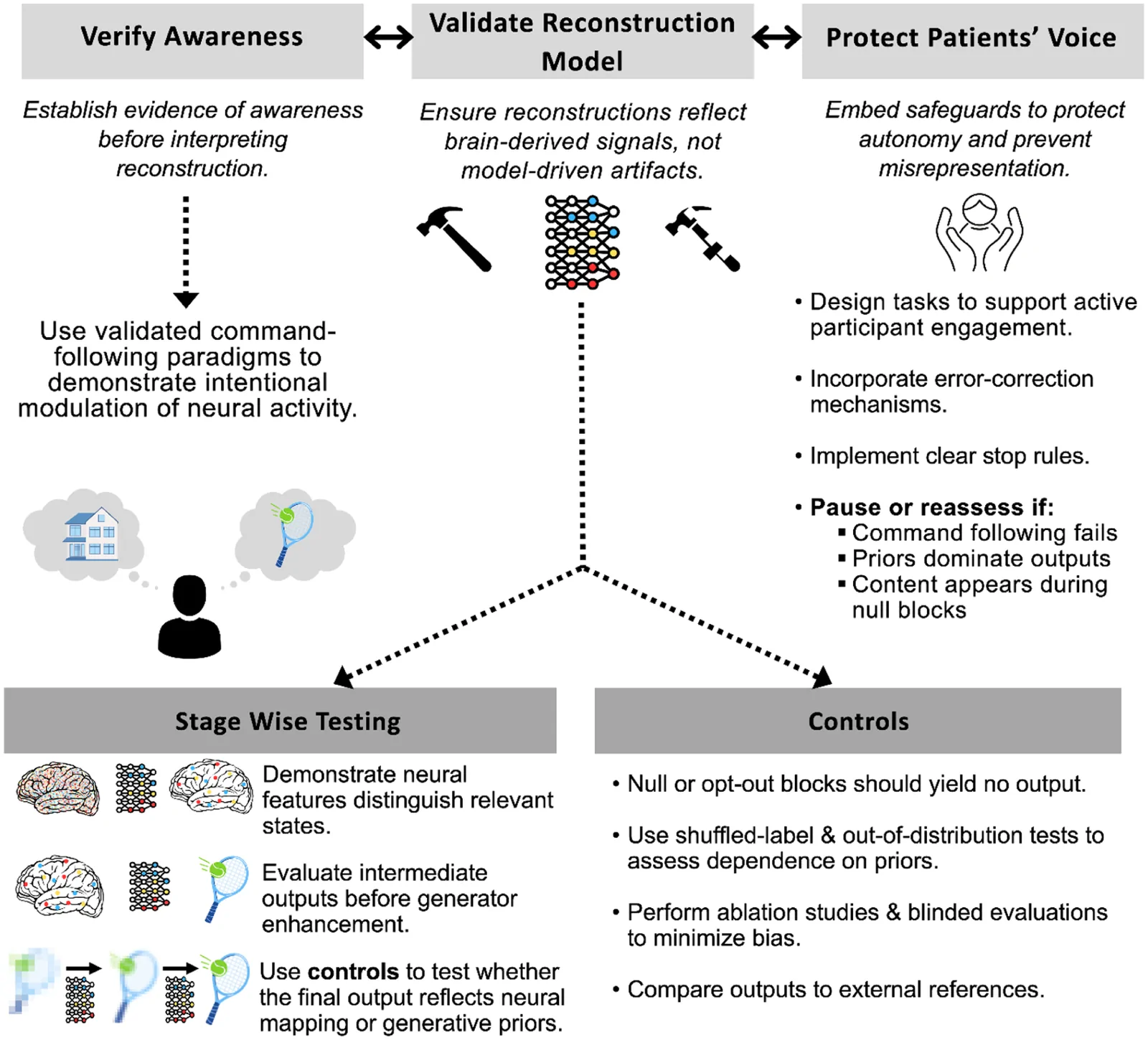
Roadmap and considerations for applying reconstruction research in patients with disorders of consciousness.

To address these concerns, a set of principles is proposed for navigating the complexities of interpreting reconstruction findings, specifically where they relate to DoCs (see Figure 5). While not exhaustive, this framework provides an initial structure to guide researchers and clinicians as reconstruction techniques become increasingly used.

### Verifying Awareness

The first step is to establish awareness. No matter how compelling a reconstruction may appear, it cannot, by itself, demonstrate conscious experience. In healthy participants, stronger evidence that reconstruction reflects subjective experience has come from demonstrations that reconstructed content generalizes beyond encoding. For example, models trained on *externally* presented stimuli have been shown to recover *internally* generated imagery and attention, as well as align with canonical perceptual illusions (Horikawa and Kamitani 2017; Shen et al 2019; Horikawa and Kamitani 2022; Cheng et al 2023). In these cases, reconstructed outputs track internally generated content rather than the physical stimulus alone, suggesting that the models capture aspects of subjective experience rather than residual encoding.

In DoCs, analogous demonstrations require evidence that reconstructed outputs depend on internal, intentional modulation of neural activity. The most reliable method for establishing awareness clinically involves validated command-following paradigms that demonstrate intentional modulation of neural activity (Owen et al 2006). These approaches are now partially supported by both European and American neurological guidelines as a means of identifying patients with covert awareness (Giacino et al 2018; Kondziella et al 2020). If neural activity changes systematically during commands but not at rest, this provides strong evidence that the patient is aware and capable of purposeful engagement.

Although command following does not resolve the philosophical concerns discussed earlier, it is difficult to conceive of a truly unaware patient consistently following instructions across repeated trials. Its adoption in guidelines, together with 2 decades of clinical and research use, provides additional justification that awareness is present and that reconstructed outputs are unlikely to reflect residual encoding alone (Owen et al 2006; Giacino et al 2018; Kondziella et al 2020; Kazazian et al 2025). Conversely, if command following cannot be demonstrated, the most appropriate conclusion is that neither awareness nor its absence has been established and that reconstruction results cannot be assumed to reflect conscious perception rather than encoding.

### Validating the Reconstruction Model

Once awareness has been established, the next priority is to ensure that reconstructions genuinely reflect neural processes rather than spurious associations introduced by the modeling pipeline. Validation should begin by isolating and testing each stage of the reconstruction process independently. When developing neural features, researchers should demonstrate that the output can reliably distinguish relevant states, such as image versus nonimage or natural scene versus face stimuli. Moreover, using different regions of interest to derive these neural features can help test hypotheses about distinctions between aware and unaware reconstructions, which can also serve as a basis for developing appropriate null models. At the transformer stage, outputs should be evaluated in their raw form before the generator refines them. This approach enables identification of which aspects of the mapping are driven by neural signal alone and which emerge only after extensive modification by downstream models.

Several different tests can detect when the model may be misrepresenting or overemphasizing the contribution at the generation stage. Wherever possible, outputs should be compared with *real-world* examples to estimate accuracy. For example, if a patient is instructed to imagine walking through their home, the resulting reconstruction could be compared to actual photographs of their home. This test should be accompanied by several statistical controls. Null or “opt-out” blocks, such as eyes-closed conditions or periods without attempted speech, should be used, during which the model should return no content. Shuffled-label tests provide another layer of control by disrupting the correspondence between stimuli and neural data, allowing researchers to estimate chance-level performance. Similarly, out-of-distribution tests, such as using distorted face configurations or nonsensical words, can reveal whether reconstructions default to priors rather than representing the specific stimulus shown to the patient. Finally, ablation studies, in which components of the model or specific input signals are systematically removed, can help determine how dependent the model is on certain features and layers.

### Protecting the Patient’s Voice

Even technically sound reconstructions can be harmful if they misrepresent a patient’s inner life or undermine autonomy. Safeguards must therefore be embedded throughout the process to ensure that patients are treated as active participants rather than passive subjects. Evidence from healthy participants demonstrates that semantic reconstruction using fMRI requires active cooperation; when engagement is withheld, meaningful reconstruction does not occur (Tang et al 2023). Although it remains unclear whether this finding generalizes across modalities or patient populations, it highlights the value of mechanisms that allow patients to indicate willingness to participate and to confirm or reject interpretations of reconstructed outputs. Error correction systems, such as the eye movement confirmation interface developed by Card et al (2024), provide one such mechanism, and secondary neural responses, such as brief imagery signals, may serve a similar role in DoC populations.

Clear stop rules are also essential to prevent harm, including circumstances in which command following cannot be reliably reproduced across sessions, model performance collapses when priors are weakened or removed, or plausible outputs emerge during null blocks or shuffled-label testing. These safeguards help prevent misrepresentation, reduce the risk of false hope among families, and limit the potential for clinicians to overinterpret ambiguous findings.

Reconstruction systems may infer perceptual or cognitive information that patients cannot choose to express or may not wish to disclose. Emerging neurotechnologies may require special legal and ethical protections, including the right to mental privacy and agency over one’s own neural information (Yuste et al 2017; Goering et al 2021). These concerns are especially salient in DoCs, where patients currently cannot provide ongoing consent or exercise control over what aspects of their internal life are accessed or communicated. Any future application of reconstruction must therefore be guided by principles that protect the boundaries of private mental experience, ensuring that the technology does not intrude upon or exploit domains of subjective life that patients themselves cannot defend.

Together, these practices offer a foundation for interpreting reconstruction results responsibly. While uncertainty will always remain, these strategies provide a pathway to distinguish genuine neural signals from model-driven artifacts and to protect patients’ autonomy and dignity. Under these conditions, reconstruction can move beyond technical achievement to become a meaningful tool for understanding and reconnecting with patients whose experiences would otherwise remain hidden.

## Conclusion

Recent advances in reconstruction, particularly in speech neuroprostheses, demonstrate that neural signals can be translated into meaningful communication, connection, and agency when cognitive and motor capacities remain intact. These developments show that reconstruction can restore functions that were once thought permanently lost and suggest genuine clinical potential for patients whose experiences are otherwise inaccessible. Applying these methods to DoCs, however, requires an additional layer of scientific humility, ethical responsibility, and procedural rigor. Compelling reconstructed outputs may reflect unconscious encoding, modeling constraints, or mappings that diverge from residual neural activity, making it essential to evaluate not only what models produce but also how these outputs are generated. This evaluation demands attention to model architecture, feature selection, stage-wise validation, and safeguards for autonomy and error correction. Realizing the scientific and clinical potential of reconstruction will depend on advances in neural signal acquisition, deep learning, interpretability, and patient-centered care, supported by systematic, individualized protocols that evaluate evidence for awareness and protect the authenticity of patients’ conscious contents. If these standards are met, reconstruction has the potential to transform neuroscience and clinical practice—recovering otherwise inaccessible experiences in those who cannot speak or move, reconnecting patients with their world, and restoring communication and dignity where none seemed possible.
